# Unexpected presence of ^14^C in inorganic pigment for an absolute dating of paintings

**DOI:** 10.1038/s41598-020-65929-7

**Published:** 2020-06-12

**Authors:** Lucile Beck, Cyrielle Messager, Ingrid Caffy, Emmanuelle Delqué-Količ, Marion Perron, Jean-Pascal Dumoulin, Christophe Moreau, Christian Degrigny, Vincent Serneels

**Affiliations:** 1 Laboratoire de Mesure du Carbone 14 (LMC14), LSCE/IPSL, CEA-CNRS-UVSQ, Université Paris-Saclay, Gif-sur-Yvette, France; 2 Haute Ecole Arc Conservation-Restauration; HES-SO // Haute Ecole Spécialisée de Suisse Occidentale, Espace de l’Europe 11, 2000 Neuchâtel, Switzerland; 3 Sarl Germolles, Château de Germolles, 100 place du 05 septembre 1944, 71640 Mellecey, France; 4 Université de Fribourg, Département de Géosciences, Chemin du Musée 6, 1700 Fribourg, Switzerland

**Keywords:** Chemistry, Mass spectrometry, Archaeology

## Abstract

The absolute dating of paintings is crucial for tackling the problem of fake art. Investigations to authenticate paintings rely on an advanced knowledge of art history and a collection of scientific techniques. Radiocarbon dating is the only technique that gives access to an absolute time scale, but its application is limited to organic materials such as wood, canvas or natural binder. Extending absolute dating to inorganic pigments would make it possible to overcome the lack of available materials for dating easel and mural paintings. Here, we present a novel technique permitting paintings that contain inorganic pigment to be radiocarbon dated. We report results obtained on lead white that was the major white pigment used from Antiquity to the 20^th^ century. We demonstrate that its manufacture is the key point for an absolute and reliable dating. We report an unprecedented use of ^14^C to date 14^th^ to 16^th^ century wall paintings. Since lead white was extensively used by the greatest artists, we anticipate that this study will open new avenues for detecting forgeries on the art market and for museums.

## Introduction

Investigations to authenticate paintings rely on an advanced knowledge of art history and a collection of scientific techniques such as X-ray radiography, multispectral imaging and chemical analysis. Only two methods give access to an absolute time scale to date these artworks: dendrochronology and radiocarbon (carbon14) dating.

Paintings consist of several superimposed layers containing two main ingredients, pigments and binder, that may or may not be covered with varnish. Some of these components are good candidates for radiocarbon dating since they originated from natural organic materials. Until the 20^th^ century, the binder was mainly made from plant or animal material, such as vegetal oils for oil painting, egg for tempera and more rarely beeswax for encaustic. However, preservation of the binder over the centuries is not always certain^1,2^ and it can be subject to modern contamination by synthetic resins used for restoration, retouching or varnishing^3^. As a result, the amount of original carbon still present can be very low, except for recent art works or forgeries^4,5^.

For easel paintings, supports such as wooden frame or panel, canvas fabric, or paperboard are suitable for radiocarbon measurement. Wood and canvas dating has provided evidence in authentication issues^5,6^, but a time lag of several years can be observed between the radiocarbon date and the date of the painting^7^. The difference is due to the time lag between the harvesting of the plants for making canvas or the felling of the trees for making the frame and completion of the artwork. It could also reflect long-term storage of the materials by the supplier or in the artist studio. Furthermore, forgers or the artists themselves may use or reuse old supports for their creations, which makes the date inconclusive

In the case of mural paintings, organic supports are not available since paint or pigments are directly applied on a mineral wall, rock, dry or wet plaster (*secco* or *fresco*). For *fresco* mural paintings, no organic binder is present since pigments are mixed with water. Thus, pigments within the paint layers are the only suitable material that can be dated by ^14^C in order to obtain absolute dating of murals.

Ancient pigments were far more frequently composed of minerals. Most of them do not contain carbon (reds, oranges, yellows, browns, and purples are iron oxide/hydroxides, lead oxide or mercury sulfide, greens and blues are copper-based alumino-silicates,…) except for some geological pigments such as copper and calcium carbonates. Until recently, only the carbon black pigment produced from wood charcoal has been dated by the radiocarbon method^8–11^. Plants contain ^14^C incorporated during their life by exchange with the atmosphere. When the trees are cut to produce charcoal, ^14^C is no longer fixed and decreases through radioactive decay with a half-life of 5700 years^12^. Thus, the measured level of ^14^C leads to an estimate of the date of death of the organism and by extension of the production of the wood charcoal. Radiocarbon dating of the black pigment of rock painting became possible with the advent of accelerator mass spectrometry (AMS) which is capable of measuring less than one milligram of carbon. AMS radiocarbon dating of charcoal black pigments from many decorated prehistoric caves around the world has provided invaluable contributions to rock art studies by revealing ancient paintings and drawings^13,14^.

Extending absolute dating to other pigments would overcome the lack of binder and the absence of organic support for dating murals and consolidate or reject questionable results obtained from the frame or the canvas for easel paintings. A technique permitting paintings that contain inorganic pigments to be radiocarbon dated would meet this need. Two widespread white pigments – calcium carbonate and lead white - are carbonate-based pigments and contain carbon. Both are considered as mineral pigments, but are produced differently. Calcium carbonate (CaCO_3_) is a natural pigment extracted from quarries. It was formed several million years ago from micro-organisms, but due to the ^14^C radioactive decay of 5700 years, geological CaCO_3_ no longer contains enough ^14^C to be radiocarbon dated. Conversely, lead white is synthesized. Lead white, composed of cerussite (PbCO_3_) and hydrocerussite (Pb_3_(CO_3_)_2_(OH)_2_), has been artificially produced by lead corrosion since Antiquity^15^. This corrosion process, involving metallic lead, vinegar and organic substances such as horse manure or tan bark, was also named the stack or Dutch process when mass production started from the 16^th^ century on. Recent studies have evidenced the role of the organic ingredients in the formation of cerussite and hydrocerussite^16,17^ and a growing interest for dating lead carbonate-based pigments by the radiocarbon method has emerged in the last two years^18,19^. Lead white was extensively used by house painters as well as by the masters Da Vinci^20–22^, Vermeer^23^, or Van Gogh^24,25^; dating this pigment thus opens new avenues for the authentication of works of art.

One of the difficulties is to separate the carbon originating from lead white without contamination from calcium carbonate pigments present in the paint sample and depleted in ^14^C. The conventional protocol based on hydrolysis to extract carbon is not selective as phosphoric acid reacts with all carbonates. To solve this issue, we developed an innovative protocol based on thermal separation. The precise control of CO_2_ release enables organic carbon from lead white to be accurately selected and collected and geological carbon from other carbonate compounds to be rejected. The advantage of this technique is that contamination by dead carbon from other pigments such as calcite is efficiently eliminated. This procedure tested on modern white pigments provides robust data, free from contamination for a reliable dating of the paint layers.

In this study, we report the unprecedented use of ^14^C to date lead white and verdigris from mural paintings, demonstrating the capabilities of the radiocarbon method for dating paintings containing inorganic pigments artificially produced according to the corrosion process. We show by dating different productions of lead white that the manufacturing process is the key to radiocarbon dating one of the major pigments used in the past. Absolute dating of medieval paintings was achieved on well-documented paintings from the Château de Germolles in Burgundy, France and on painting fragments recently discovered in the church of the Cordeliers in Fribourg, Switzerland.

## Results

### Efficient carbon source selection by thermal decomposition

A thermal protocol was applied to prepare lead white samples for radiocarbon dating. Prior studies were carried out to adjust the heating temperature. By controlling the CO_2_ release, it was possible to monitor the amount of carbon extracted and to select the best conditions to collect carbon from lead white without taking the risk of extracting exogenous carbon^26^. A temperature of 400 °C, which is relatively low in comparison with the conventional preparation, was determined to decompose lead carbonates. This protocol was used to extract carbon from white pigments (see Method) and the results are reported in Table 1. For lead white pigment composed of pure cerussite (sample LW(YS)), 1.1 mg of C was collected for an initial mass of pigment of 25.13 mg. The ratio between these two masses corresponds to the percentage of carbon totally extracted. For sample LW(YS)), this value of 4.39% indicates that about 98% of the carbon contained in cerussite was collected. For lead whites composed of a mixture of cerussite and hydocerussite (samples LW(HM)), 3 to 3.5% of C was extracted corresponding to a ratio of about 1:3 cerussite-hydrocerussite, typical of lead white pigment obtained by the historical corrosion process using natural fermentation^27^. In all cases, the carbon content was fully extracted, making it possible to reduce the initial sample size to a few mg. We prepared pure calcite (C) and a mixture of lead white and calcite (LW-C) pigments as for pure lead white. For pure calcite, no measurable quantity of CO_2_ was detected, indicating that less than 0.005 mg of carbon was extracted from calcite at 400 °C. In the case of lead white and calcite mixture (LW(YS)-C), the percentage of carbon extracted, 3.07%, corresponds to the amount of carbon present in lead white only (3.17%). Therefore, in both cases, no detectable carbon was collected from calcite, whether from the pure geological sample or from the mixture. The detection limit of C being 0.005 mg, we can assume that the maximum contamination possible by 10 mg of calcite would be 0.05% in the percentage of extracted carbon. At this level, we can consider that the thermal preparation of lead white pigments, based on thermal decomposition at 400 °C, is effective with a yield close to 100% and very selective for lead carbonates, providing samples free of dead carbon contamination. This step is crucial for a reliable radiocarbon date.

**Table 1:** Percentages of carbon extracted from lead white (LW) prepared by the corrosion process (with yeast (YS) or horse manure (HM)), calcite (C) and a mixture of the two pigments (LW(YS) - C) at 400 °C in vacuum.

Sample	Pigment	Production mode	Composition	Initial mass of lead white (mg) (± 0.01 mg)	Initial mass of calcite (mg) (± 0.01 mg)	Mass of carbon extracted (mg) LOD = 0.005 mg	Total %C extracted	Expected Total %C extracted
LW(YS)	Lead white	Corrosion process using yeast and sugar	Cerussite	25.13	–	1.1	4.39	4.49
C	Calcite	Natural mineral	Calcite	0	10.51	No gas detected <0.005	No gas detected <0.05	0
LW(YS) - C	Lead white and calcite	Various (see above)	Cerussite (71%) and calcite (29%)	25.46	10.40	1.1	3.07	3.19
LW(HM)-1	Lead white	Corrosion process using horse manure	Cerussite + Hydrocerussite	27.07	–	0.94	3.48	unknown
LW(HM)-2				9.65	–	0.32	3.31	unknown
LW(HM)-3				5.90	–	0.18	3.05	unknown

(LOD = limit of detection).

### Corrosion process of lead is the key for radiocarbon dating of lead white pigment

To test the hypothesis that the production route of lead white pigment is the criterion for the application of radiocarbon dating, we report here results obtained on modern pigments prepared with various processes (Table 2). The year or the period of production were known from the suppliers.

**Table 2:** Radiocarbon measurements of modern lead white pigments prepared according to different production processes.

Sample	Pigment/Composition	Production mode	Fermentation environment	Date of production	pMC	Radiocarbon date (calAD)	Lab ref N°
LW(YS)	Lead white Cerussite	Corrosion	Yeast and sugar	2015	102,03 ± 0,22	1955–1956 (22.6%) 2013–2016 (77.4%)	SacA52822
LW(HM)-2	Lead white Cerussite + Hydrocerussite	Corrosion	Horse manure	2016	101,70 ± 0,30	1955–1956 (28.2%) 2013–2016 (71.8%)	SacA51770
LW(I)	Lead carbonates	Industrial	No	≈2000–2010	6.44 ± 0.14	22030 ± 180	Sac 47186
LW(Min)	Cerussite	Natural mineral	No	-	0.70 ± 0.04	39840 ± 490	SacA49227

For the samples prepared according to the corrosion process, the results are in agreement with the date of production. Calibration of the dates was obtained from the bomb peak calibration curve showing two probable age distributions due to the shape of the curve (Fig. S1). The 2013–2016 interval coincided perfectly with the dates of production. This result is direct evidence that during the synthesis, atmospheric/organic carbon was incorporated into the lead carbonate compounds. Carbon thus carries the ^14^C signature contemporaneous of the production, demonstrating that CO_2_ released by the fermentation of the horse manure or the sugar-yeast preparation is a reactant of importance for the formation of lead carbonates when lead corrodes. Organic CO_2_ interacts with lead to form hydrocerussite (Pb_3_(CO_3_)_2_(OH)_2_) and cerussite (PbCO_3_). As a result ^14^C is present in both compounds whatever their ratio, and both can be dated by the radiocarbon method.

On the other hand, when lead carbonate was produced from an industrial process based on wet chemistry or collected as a natural mineral, the ^14^C content is very low, producing very old apparent ages. Most of the chemicals derive from fossil sources and, like geological minerals, they contain almost no ^14^C due to the short radioactive decay of 5700 years.

These results show that ^14^C measurements provide a robust criterion to distinguish traditional lead white pigment resulting from a synthesis by corrosion from those obtained by industrial routes or isolated from a natural source. They also demonstrate the strong potential of the radiocarbon method to date lead white based historical paintings.

### Dating inorganic pigment in medieval wall paintings

**Figure 1:**
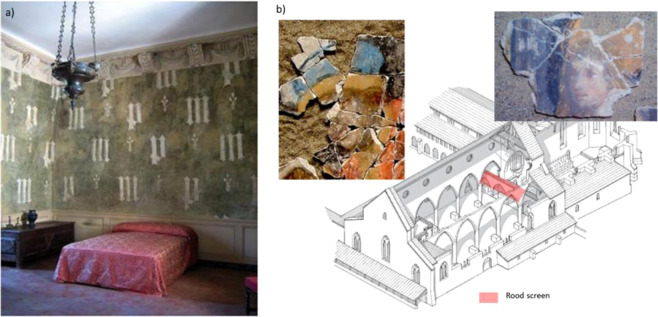
Medieval sites with the paintings selected for radiocarbon dating of lead white. (**A**) Marguerite of Bavaria’s dressing room in the Château de Germolles, Burgundy, France (photo: E. de Lavergne), (**B**) The restitution of the destroyed rood screen in the Church of the Cordeliers, Fribourg, Switzerland (in red)^51^ and some fragments of the wall painting discovered during excavation.

We report the results obtained on two well-preserved medieval wall paintings (Fig. 1). The first painting is the courtly decoration of Margaret of Bavaria’s dressing room at the Château de Germolles, Burgundy, France dated 1388–1390. The second group of paintings comes from the decorations of the destroyed rood screen of the Church of the Cordeliers in Fribourg, Switzerland. Two groups of paintings were uncovered. Based on stylistic attribution, the main artwork was dated 1500–1510, whereas the second decoration remained inaccurately dated, with dates between 1340 and 1700 being proposed (for more information, see Materials and Methods). We select the paint fragments on the basis of their known historical context and chemical composition^28,29^. The pigments are composed of pure lead white or lead white associated to green copper-based pigment. We prepared nine fragments of lead white (Figures S2 and S3) by the thermal procedure and we performed the carbon isotope measurements by AMS (see Method). For comparison, we also dated 3 samples painted with charcoal black (carbon pigment).

**Figure 2:**
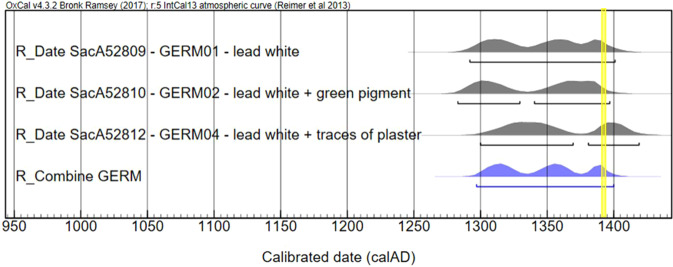
Consistency of the calibrated ages for lead white samples from the wall paintings at Château de Germolles, Burgundy, France. Calibrated radiocarbon dates of lead white-based paints are represented in grey and the statistical combination of the three dates in blue (1297–1400 AD). A χ² test value of T = 2.1 (5% 6.0) shows the consistency of the dates. The yellow line represents the expected date (1388–1390).

The dating results obtained for the wall paintings of the Château de Germolles are reported in Table 3. The radiocarbon dates of three samples, GERM01, GERM02 and GERM04 are 1292–1401 AD, 1283–1397 AD, and 1300–1419 AD, respectively. The statistical combination is reported in Fig. 2. The ages of the three samples are consistent according to the statistical χ² test of the OxCal 4.3 software (T = 2.1 (5% 6.0))^30,31^. The presence of a green copper-based pigment, probably copper acetate (verdigris) in addition to lead white in the sample GERM02 does not seem to significantly influence the result. As verdigris was produced in the same way as lead white (see the recipe of Pierre de Saint Audemar, in the Discussion section), we assume that this green pigment also contains carbon of organic origin.

**Table 3:** Radiocarbon dating results of lead white paint layers from the wall paintings at Château de Germolles, Burgundy, France.

Laboratory reference	Samples	Pigment composition	^14^C age ± 1σ (year BP)	Calibrated age range (95.4%)
SacA 52809	GERM01	lead white	620 ± 30	1292–1401
SacA 52812	GERM04	Lead white and traces of plaster	580 ± 30	1300–1369 (63.6%) 1381–1419 (31.8%)
SacA 52810	GERM02	lead white + green copper-based pigment	640 ± 30	1283–1329 (41%) 1340–1397 (54.4%)

The combination of the dates gives the interval 1297–1400 AD with three periods of higher probability at around 1315, 1355 and 1390 years AD. The age distribution can be refined knowing that the castle was purchased in 1380 (see Methods). This constraint was introduced in the modelling sequence of Oxcal 4.3^31^, leading to the reduced age distribution 1380–1400 AD (Fig. S4). This interval is in perfect agreement with the report in the years 1388–1390 of the decoration work in the castle accounts^32^.

The dating results obtained for nine fragments of the destroyed rood screen of the Church of the Cordeliers in Fribourg, Switzerland are reported in Table 4. Six fragments come from the main artwork (decorations 1 to 4) and the other three are from a second painting (decoration 5).

**Table 4:** Radiocarbon dating results of lead white and black paint fragments from the rood screen in the Church of the Cordeliers, Fribourg, Switzerland.

	Laboratory reference	Samples	Pigment composition	Radiocarbon date ± 1σ (years BP)	Calibrated date (95.4%)
Decorations 1 to 4	SacA54647	FRIB14	lead white	470 ± 30	1410–1457
	SacA54650	FRIB17	lead white	405 ± 30	1433–1522 (79.1%) 1575–1624 (16.3%)
	SacA54651	FRIB18	lead white	450 ± 30	1415–1479
	SacA54649	FRIB16	lead white (micro-sample)	550 ± 100	1262–1522 (91.8%) 1575–1625 (3.6%)
	Sac56376	FRIB19	Black charcoal	440 ± 30	1417–1490 (94%)
	Sac56377	FRIB110	Black charcoal	450 ± 50	1398–1523 (84.4%) 1572–1630 (11%)
Decoration 5	SacA54652	FRIB52	lead white	410 ± 30	1430–1522 (82.8%) 1578–1583 (0.5%) 1591–1620 (12.1%)
	SacA54653	FRIB53	lead white (micro-sample)	295 ± 45	1472–1666 (93.7%) 1785–1795 (1.7%)
	SacA52837	FRIB51	Black charcoal	310 ± 30	1485–1650 (93.7%)

**Figure 3:**
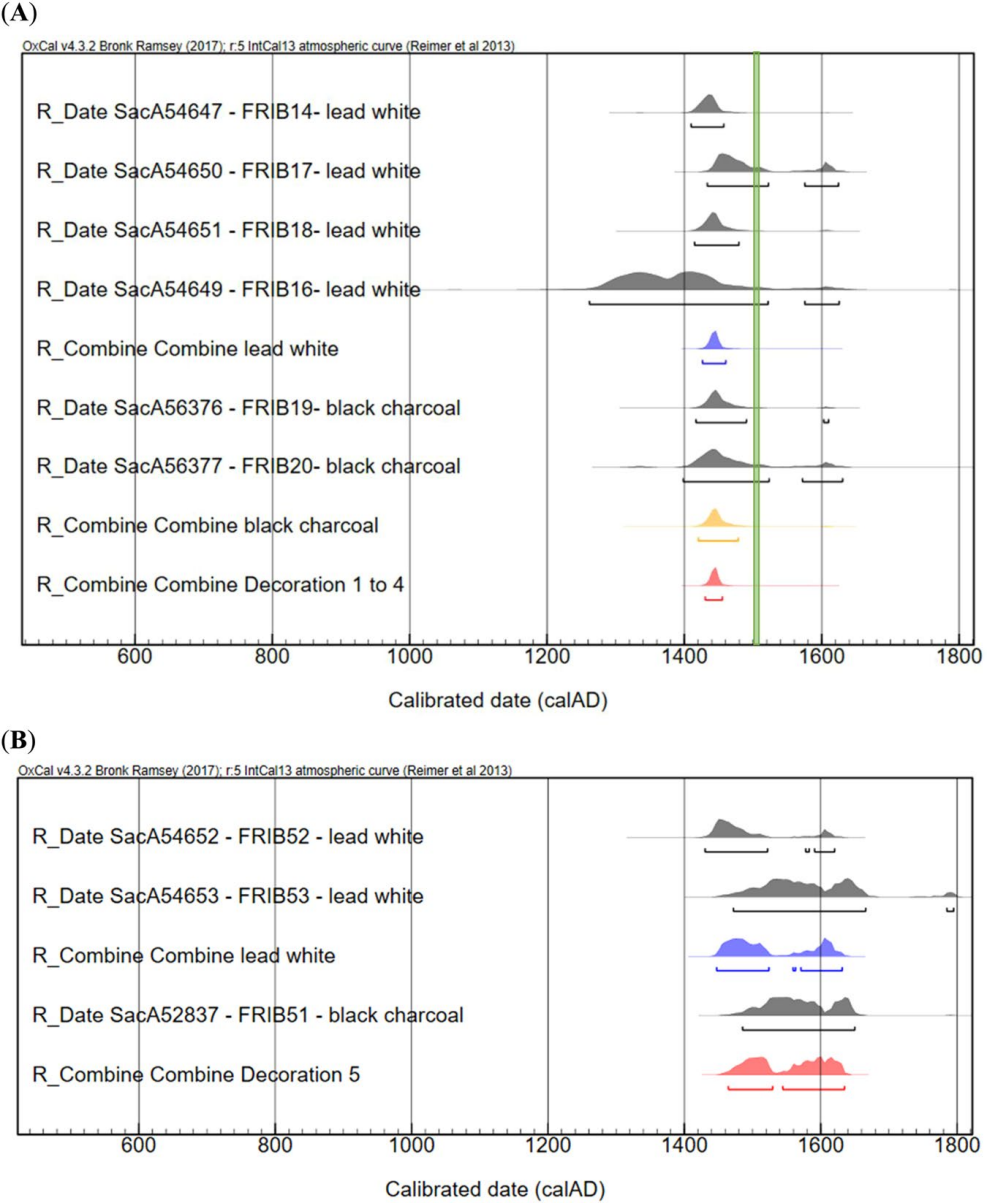
Calibrated ages for lead white and charcoal black paint layers from the decorations at the Church of the Cordeliers. Age probability distributions are represented in grey. (**A**) For decorations 1 to 4, the combination of the dates is 1426–1460 calAD for the lead white paint samples (in blue), 1420–1478 calAD for the charcoal black paint samples (in orange) and 1430–1455 calAD for all the samples (in red). The green line represents the expected date (1500–1510). (**B**) For decoration 5, the combination of the dates is 1447–1631 calAD for the lead white samples (in blue). Combination of lead white dating with charcoal black dating gives 1464–1635 calAD (in red).

The radiocarbon dating results of decorations 1 to 4 are from 1262 to 1630 AD (Fig. 3a). The statistical combination of the lead white samples is consistent with a χ² test value of T = 3.6 (5% 7.8) and the combined result of the dates is 1426–1460 AD. The combined result obtained for the black paint layers is very close, with a date interval of 1420–1478 AD. Both groups of dates are consistent and the combined result gives the range 1430–1455 AD for both white and black pigments.

These similar results between lead white and charcoal black provide direct evidence that lead white can be accurately dated by the radiocarbon method. However, the paintings are attributed to the official painter Hans Fries present in Fribourg between 1490–1510^29^. A shift of about 50 years older than this expected date is observed for both pigments. It should be pointed out that the radiocarbon dating of lead white is related to the date of manufacture of the pigment and not to the execution of the painting. The difference of 50 years may be related to the pigment trade market and distribution in Europe in the 16^th^ century. As it was an important pigment for house paints, lead white was produced in large quantities^16^ and may have been maintained in storage for a long period. The radiocarbon dating of charcoal black may also be affected by the inbuilt effect, which may result from the growth of the tree or the storage between the death of the tree and its use as charcoal pigment^33^. But since all the dates fully agree, we can also assume that other factors relative to the fluctuations of the calibration curve as well as the statistical combination induce a bias. The attribution of the paintings could also be re-examined. At this stage of the investigation, it is not possible to decide between these assumptions but we can conclude that the radiocarbon results on lead whites are reliable when compared to charcoal dates. The dates are overall in accordance with the expected period.

The radiocarbon dating results obtained on the paint layers of decoration 5 are from 1430 to 1666 AD (Fig. 3b). This interval is very close to the result obtained on a black carbon pigment coming from the same decoration (1485–1650 AD). Although somewhat large, this range nevertheless gives more precise dates for the execution of the painting than the interval of 1340 to 1700 proposed by the curators. The results suggest that decoration 5 was created shortly after decorations 1 to 4. However, the paintings cannot be assigned to Hans Fries from the stylistic and technical points of view. This part of the decoration is doubtless the work of another artist, probably covering another part of the rood screen.

## Discussion

In this study, we demonstrate that it is possible to extract all the carbon from lead carbonates by thermal decomposition in order to date lead white pigments and paintings by the radiocarbon method. The synthesis process of lead white is identified according to the ^14^C content and we show that the ^14^C isotope is therefore an important marker for reconstructing the history of lead white production. The process most commonly used over time is the corrosion process occurring in a fermenting environment. Metallic lead and vinegar were placed in pots embedded in horse manure or other organic substances. By fermentation, organic substances degrade and release CO_2_ that carries ^14^C. The organic substance are thus the key component to obtain a reliable and absolute radiocarbon date. Medieval paintings are a perfect example showing that recipes reported in historical manuscripts were applied. The radiocarbon measurements date the pigment production and provides new insights into the creation of the wall paintings.

However, some points of the recipes found in ancient and medieval manuals still need clarification. A fermenting environment is not always reported as in Theophrastus, *Liber de lapidibus*^34^, or in Isidore of Sevilla, *Ethymologies*^35^. This ingredient appeared in the historical documentation from the 13th and the 14th century onwards under the term of horse manure. It is also questionable whether the container should be hermetically sealed and then covered with horse manure. It seems difficult to explain this recommendation of the medieval authors as it would prevent the incorporation of CO_2_ unless the jar is sufficiently porous. Thus, Pierre of S. Audemar wrote in *De Coloribus Facendis*:^36^

*152. How to make and temper white and green.–White and green colours, without salt, are made and tempered as follows:****Pour very strong vinegar into a vase****, and place twigs of trees across it inside the vase, and then place****strips of lead****, and other strips of copper or brass, suspended in the air by means of the twigs, so as not to touch the vinegar or each other. Then****close the vase****very carefully, and****lute it with clay or cement, or wax****, so that there may not be the least hole through which the vinegar may exhale.****Then cover it with horse-dung****, and, after****30 days****, on account of the acidity of the vinegar or the wine – for the wine, on account of the heat of the dung, will become vinegar- on account, I say, of the acidity of the wine or vinegar,****the copper or brass will be found to be turned green and the lead white****. Take the white, dry it, and grind it, and temper it with wine, and use it for painting on parchment, and mix it with oil for painting on wood and on walls. In the same manner grind and temper the green with oil, and use it for painting on wood; but on walls with wine, or, if you prefer it, with oil. On parchment, however, you must not grind it with oil, but you must temper it with very clear and good wine, or with vinegar.*

In the 15^th^ century, an anonymous Italian author compiled recipes to manufacture and prepare colors for paint in his book *Segreti per colori*^37^. At that time, Venice was a major center for the production of lead white. He described the manufacture of this pigment very clearly. This recipe, which dates back to Venice’s hegemony on the pigment market, was to serve as a model for the production of lead white.

*195. To make white lead.–****Take leaden plates****, and suspend them over the* ***vapour of very strong vinegar in a vase***, ***which after being heated must be placed in dung for two months;*** *then scrape away the matter that you will find upon the plates, which is the white lead. Do this until the plates are consumed.*

Thus, the results obtained on the two medieval wall paintings are in agreement with the recipes published in the 14^th^ and 15^th^ century. This traditional recipe (using metallic lead, vinegar and horse manure) continued to be used until the very beginning of the 20^th^ century with some variations. Some sources mention the replacement of horse manure by spent tanning bark (the stack process prior to the 18^th^ century) or fermenting wine lees (the German or chamber process in the late 18^th^ century)^38,39^. But these modifications should not alter radiocarbon dating of lead white as all these materials are organic compounds.

Consequently, the reaction mechanism recently proposed for the synthesis of lead white^17^ is confirmed here: in a first step, the action of vinegar on metallic lead in the presence of dioxygen enables the formation of a lead acetate (Pb_4_O(CH_3_COO)_6_). In the second step, carbon dioxide released by the fermentation of the horse manure or the tan bark acts to transform lead acetate into plumbonacrite then hydrocerussite and cerussite. Depending on the amount of carbon dioxide, the final products may vary from hydrocerussite to cerussite, but both have trapped CO_2_ produced by the degradation of the organic substances. The two lead carbonates thus contain carbon 14, making radiocarbon dating of the lead white pigment possible.

In addition, in painting manuals, some authors such as Theophrastus or Pierre of Saint Audemar note that green copper pigment is synthesized in the same way as cerussite/lead white. This point is confirmed in this study by successfully dating a sample containing both pigments. Hence, the radiocarbon dating method can be extended to other synthesized pigments such as copper carbonates.

From the 19^th^ century on, new synthesis processes were developed to improve yields and lower production costs. Cerusse factories introduced innovations in their facilities and other CO_2_ production methods were used, such as coal fires or the addition of marble or potassium carbonate to the vinegar. Wet processes such as the Clichy process, which relied on lead white precipitation, were invented and completely turned away from the ancient recipes^40^. The development of these methods prevents the radiocarbon dating demonstrated in this study since organic CO_2_ sources were no longer used and new CO_2_ sources made from fossil carbon are free of ^14^C. However, the radiocarbon measurements can then complement structural analysis^27^ to detect pigments manufactured in the 19^th^ and 20^th^ century.

In summary, the methodology developed here can be applied to any archaeological objects or works of art made of carbonates synthetized by the corrosion process. Cosmetics and paintings produced from Antiquity to the 19^th^ century and that contain lead white can now be dated by the radiocarbon method. However, there are some limitations. In the case of more complex mixtures of paints, and in particular in presence of other carbonates or recent acrylic resins, a prior analysis of the constituents is recommended so as to adapt the extraction protocol in order to prevent any contamination from geological or fossil carbon. The preservation of the painting has to be carefully examined before sampling to select original paint layers far from restoration areas. However, the alteration of paint layers, or the presence of natural binders derived from plant or animal material, should not lead to biases insofar as the materials involved are contemporaneous of the lead white production^41^. Finally, the sample size is a compromise between the preservation of the artwork and the precision of the dating result. AMS facilities make it possible to analyze low amounts of carbon (0.1 to 1 mg in routine) which corresponds to 5 to 25 mg of lead white. Smaller samples up to a few micrograms C can be dated with gas source facilities or/and appropriate data processing^19,42,43^, but the measurement uncertainty is larger and the risk of contamination greater.

## Conclusion

Absolute dating of paintings that contain inorganic pigments has been achieved. The successful dating of lead white in medieval paintings confirms that the radiocarbon dating method can be extended to inorganic paint materials such as synthesized carbonate pigments.

The radiocarbon dating of lead white is innovative and opens new perspectives. In the field of the history of techniques, the characterization and dating of lead white makes it possible to investigate ancient recipes of pigments and asserts the validity of these historical sources in extending our knowledge of the early steps of chemistry. ^14^C is an efficient marker to distinguish an ancient lead white manufactured by corrosion from lead whites manufactured by modern processes. This study also reveals the possibility of dating micro-samples or samples containing very small amounts of lead white. Reducing the sample size is an essential prerequisite for the application of the method to valuable paintings. In the field of art history and forensic sciences, dating lead white pigment provides a new tool for dating paintings. Since lead white was extensively used by the greatest artists, Da Vinci, Vermeer, Van Gogh and many others, we anticipate that this study will be a starting point for developing new approaches to authenticate paintings and detect forgeries. The impact for the art market and for museums is considerable.

## Method

### Experimental design

Non-restored medieval buildings were prospected in order to collect original lead white samples from paintings. Two places with untouched wall paintings were found: the medieval castle Château de Germolles in Burgundy, France and the church of the Cordeliers in Fribourg, Switzerland.

The wall painting of Germolles represents a courtly set composed of the initials M and P intermingled with thistles (Fig. 1a). Philippe the Bold, Duke of Burgundy, purchased the castle in 1380 then offered it to his wife, Margaret of Flanders, the year after. She intended to convert this fortress into a princely palace and asked Jean de Beaumetz, the official painter of the Duke of Burgundy’s Court, to take care of the castle wall decorations. The wall decoration under consideration is the one intended for the daughter-in-law’s (Margaret of Bavaria) dressing room. Thanks to the accounting notes describing the thistle decorations applied on a green background, we know that this work was executed between 1388 and 1390^32,44^. X-ray Fluorescence Spectroscopy identified lead on the white M and P and copper on the green background. Both white and green paintings were sampled: GERM01 (SacA52809) and GERM02 (SacA52810) (Fig. S1). In the 19^th^ century, the medieval paintings were covered with plaster. They were rediscovered by chance during World War II, partly cleaned of their 19^th^ century plaster and restored between 1989 and 1994. The original decoration was preserved on the back of one plaster fragment that fell down in past years. The remaining painting was collected: GERM04 (SacA52812) (Fig. S2).

During the archaeological excavations inside the Church of the Cordeliers in Fribourg, Switzerland, about 40,000 fragments of painted plasters were recovered under the modern floor. They once belonged to the painted decoration of the nave of the church, in particular the rood screen build to separate the nave and the choir. From various historical sources, it is known that the rood screen was erected around 1300 and destroyed in 1745^45^. The remnants of a remarkable artwork (decorations 1 to 4) were investigated by a multidisciplinary team as part of a research project supported by the Swiss National Science Foundation^45^. The material characterization of the pigments evidenced the use of lead white. The stylistic study led to the identification of the artist as Hans Fries, who was the official painter of the town of Fribourg between 1500 and 1510^29^. The reconstruction demonstrated that the painting by Hans Fries once covered the upper part of the rood screen 20.5 m wide and 1.6 m high. A second group of fragments (decoration 5) is inaccurately dated, with dates between 1340 and 1700 being proposed. The painting technique is different from decorations 1 to 4 and other pigments were used. The remaining fragments are stored at the Service Archéologique de l’Etat de Fribourg, which made the samples available for our study. The corpus of samples is composed of six fragments containing pure lead white: FRIB14 (SacA54647), FRIB16 (SacA54649), FRIB17 (SacA54650), FRIB18 (SacA54651), FRIB52 (SacA54652) and FRIB53 (SacA54653) (Fig. S3) and three fragments of black charcoal FRIB19 (SacA56376), FRIB110 (SacA56377) and FRIB51 (SacA52837).

The samples from Germolles were previously characterized by X-ray Fluorescence Spectroscopy (XRF), Laser Induced Breakdown Spectroscopy (LIBS) and Fourier-transform infrared spectroscopy (FTIR); Scanning Electron Microscopy (SEM)-EDS investigation was carried out on a small flake taken from the wall^28,46^. Artworks from Fribourg were characterized by X-ray Diffraction (XRD), XRF, and SEM^45^ (Table S2).

Modern lead whites were obtained from a supplier who reproduces or adapts historical processes.

### Lead white preparation for AMS ^14^C analysis

We collected between 5 and 30 mg of painting containing pure lead white or lead white associated to green copper-based pigment.

- *Sample preparation: CO*_*2*_ *collection*
  The samples were prepared using thermal decomposition of lead carbonates^26^. The samples were decomposed in vacuum at 400 °C for 1 hour to produce the CO_2_. Once the decomposition was completed, the gas was purified using two traps, one for water (mixture of ethanol/dry ice) and the other for CO_2_ (liquid nitrogen). The pressure of the purified gas was then measured and in a final step, CO_2_ was collected in a sealed tube^47^. The details of the results following the extraction protocol are summarized in Tables S1 and S3.
- *Graphitization of the sample*
  Graphitization consists of a reduction of the CO_2_ into graphite. The reduction of CO_2_ takes place at a temperature of 600 °C with hydrogen (ratio H_2_/CO_2_ of 2.5) on a catalyst iron powder. For conventional samples (mass of carbon> 0.15 mg) the mass of iron is 3 times the expected mass of carbon^48^. As the reaction is reversible, a cryogenic trap (maintained at −70 °C) is used to remove the water produced during the reaction.
  For micro-samples (mass of carbon less than 0.15 mg), a dedicated line using smaller reactors was used^42^. Magnesium perchlorate replaced the cryogenic trap. The amount of catalyst iron powder no longer depended on the mass of carbon but the quantity was fixed at 1.5 mg.
  Both facilities were used for this study depending on the mass of carbon extracted from each sample (Table S3). The graphite samples were then pressed in the target.
- *AMS Radiocarbon measurement*
  Carbon isotopes were measured with the AMS LMC14/ARTEMIS facility (Saclay, France)^43,49^. The spectrometer was adjusted according to the carbon mass. For small samples, the number of runs was increased but the duration of each was reduced^50^. Oxalic acid II was used for normalization, international intercomparison samples (FIRI H and FIRI I) for calibration, and C1 for blanks. The data were calibrated with INTCAL13^30^ using OxCal 4.3 software^31^.

## Supplementary information

Supplementary Information.
