# Supplementary material for: Unexpected presence of 14C in inorganic pigment for an absolute dating of paintings
Source: Sci Rep. 2020 Jun 12;10:9582. doi: 10.1038/s41598-020-65929-7 (PMC7293340; doi:10.1038/s41598-020-65929-7)
Supplement: Supplementary file 1 — Supplementary Information. [file 41598_2020_65929_MOESM1_ESM.pdf]

Unexpected presence of ^{14}C in inorganic pigment for an absolute dating of paintings

Lucile BECK[1*], Cyrielle MESSAGER[1], Ingrid CAFFY[1], Emmanuelle DELQUÉ-KOLIČ[1], Marion PERRON[1], Jean-Pascal DUMOULIN[1], Christophe MOREAU[1], Christian DEGRIGNY[2,3], Vincent SERNEELS[4]

[1]Laboratoire de Mesure du Carbone 14 (LMC14), LSCE/IPSL, CEA-CNRS-UVSQ, Université Paris-Saclay, Gif-sur-Yvette, France

[2] Haute École Arc, Conservation-restauration, Espace de l’Europe 11, 2000 Neuchâtel, Switzerland

[3] Sarl Germolles, Château de Germolles, 100 place du 05 septembre 1944, 71640 Mellecey, France

[4]Université de Fribourg, Département de Géosciences, Chemin du Musée 6, 1700 Fribourg, Switzerland

*lucile.beck@cea.fr

Supplementary Information

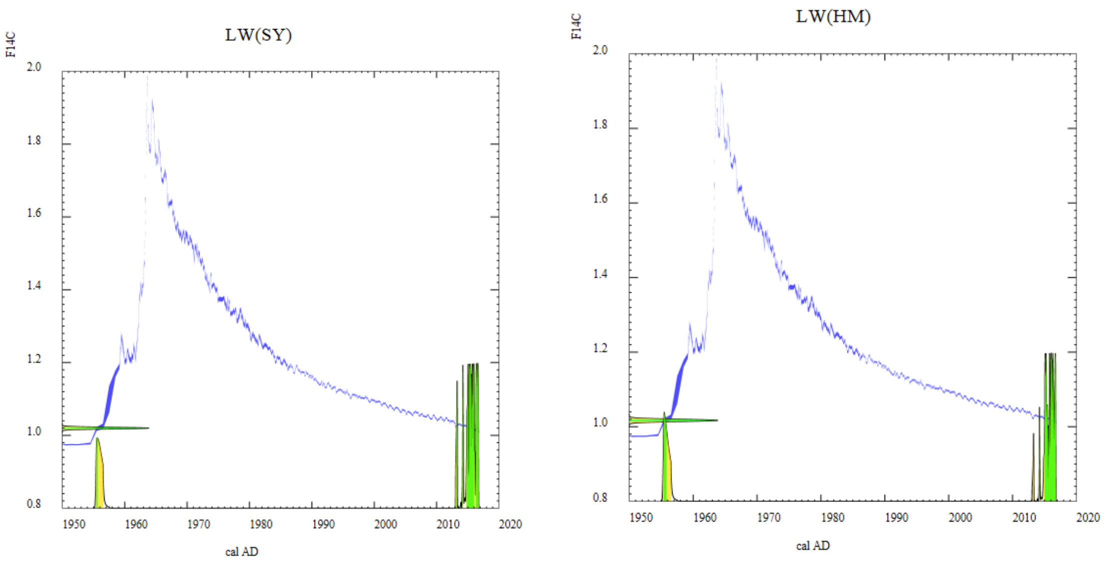

Fig. S1; Calibrated radiocarbon dates of modern lead white pigments

Calibration curve: I. Levin and B. Kromer, "The tropospheric $^{14}CO_2$ level in mid latitudes of the Northern Hemisphere" (2004) Radiocarbon 46(3):1261-1272.

Hammer, Samuel; Levin, Ingeborg, 2017, "Monthly mean atmospheric D14CO_2 at Jungfraujoch and Schauinsland from 1986 to 2016", http://dx.doi.org/10.11588/data/10100

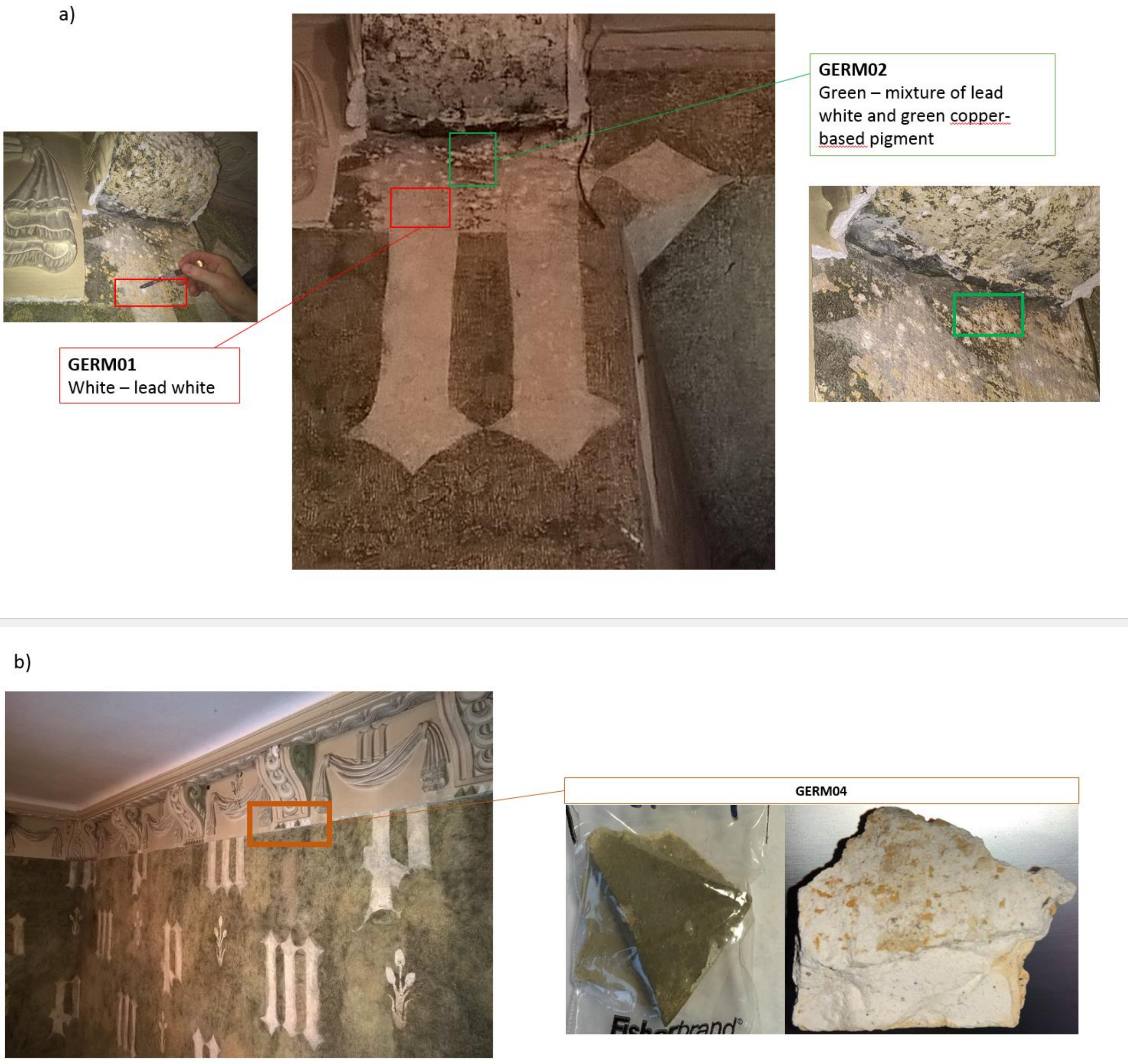

Fig. S2. Sampling in the Château de Germolles. (**A**) samples GERM01 and GERM02 were taken directly from the wall painting on an unrestored area (**B**) GERM04 was taken from one plaster triangle constituting part of the moulding, with lead white on its back side.

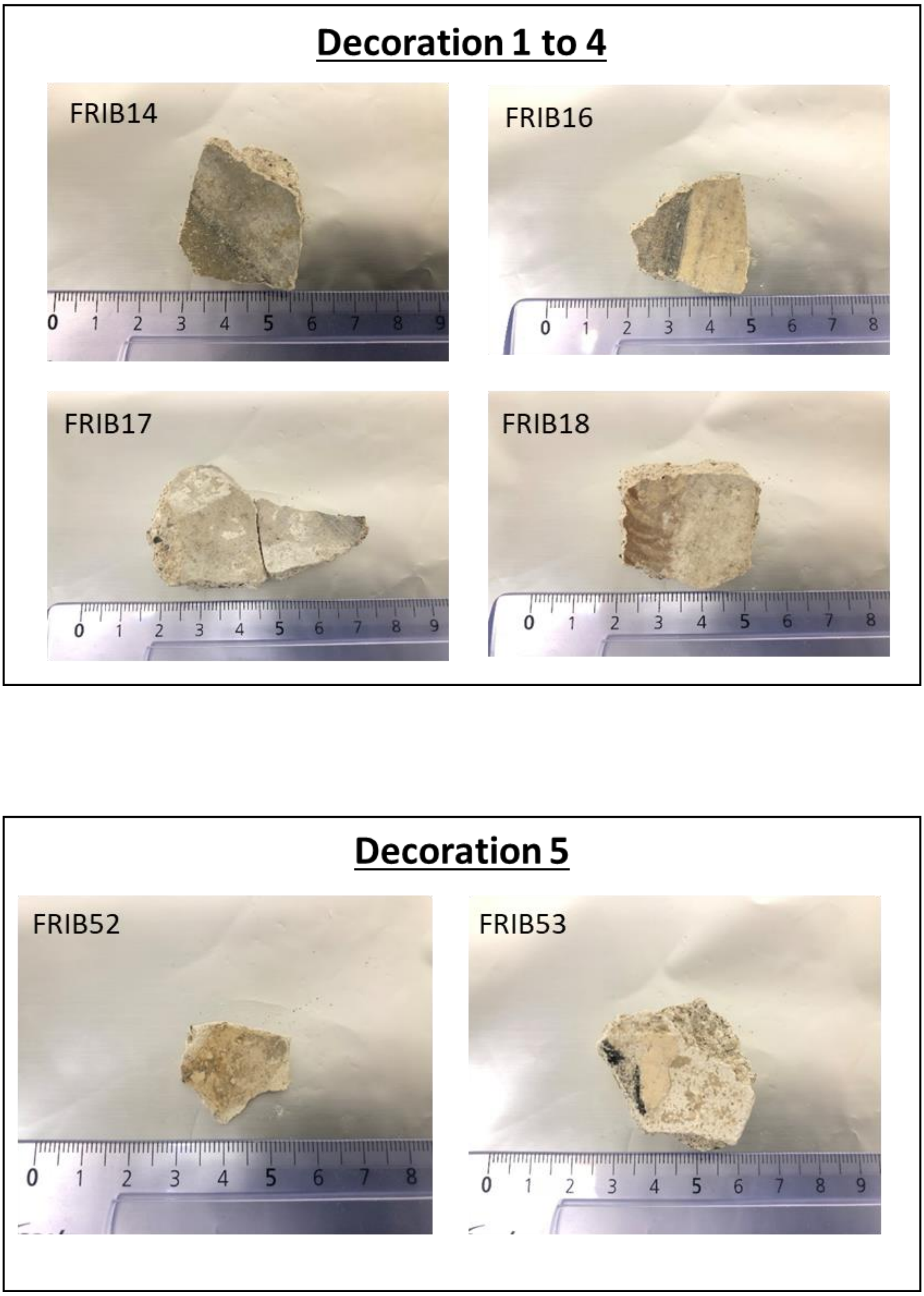

Fig. S3. White painting samples from the Church of the Cordeliers. Fragments of the rood screen from decorations 1 to 4 and decoration 5.

Table S1. Details about the quantities of carbon extracted from different manufactures of lead white, calcite and a mixture of both pigments at 400°C in vacuum

Sample	Pigment	Production mode	Composition	Initial mass of sample (mg)	Initial mass of LW (mg)	Initial mass of calcite (mg)	CO_2 collected (mbar)	Mass of carbon extracted (mg)	Total %C extracted	Expected Total %C extracted	%C extracted from LW	Expected %C extracted from LW	%C extracted from calcite
**LW(YS)**	Lead white	Corrosion process using yeast and sugar	Cerussite	25.13	25.13	-	392.9	1.1	4.39	4.49	4.39	4.49	-
**C**	Calcite	Natural mineral	Calcite	10.51	0	10.51	8.8	0	0	0	-	0	0
**LW(YS) - C**	Lead white and calcite	Various	Cerussite (71%) and calcite (29%)	35.86	25.46	10.40	392.8	1.1	3.07	3.19	4.34	4.49	0
**LW(HM) -1**	Lead white	Corrosion process using horse manure	Cerussite + Hydrocerussite	27.07	27.07	-	336.7	0.94	3.48		3.48		-
**LW(HM) -2**				9.65	9.65	-	120.8	0.32	3.31		3.31		-
**LW(HM) -3**				5.90	5.90	-	75.06	0.18	3.05		3.05		-

Table S2. Composition of the white painting samples from the Church of the Cordeliers.

Lab #	SacA54647	SacA54649	SacA54650	SacA54651	SacA54652	SacA54653
Samples	FRIB14	FRIB16	FRIB17	FRIB18	FRIB52	FRIB53
Sample details and pigments	Grey/white – lead white	Smooth white – lead white – low amount Pb	White – lead white	White – lead white	White – lead white	White – lead white – low amount Pb

Table S3. Results of the thermal decomposition of lead carbonates on medieval wall paintings.

Summary of results of the thermal extraction of carbon from lead carbonates: this table gives a short description of the characterization of each sample and compares the mass of sample prepared to the mass of carbon extracted giving the %C extracted.

N°SacA	Sample	Analytical Characterization	Initial mass of sample (mg)	CO_2 collected (mbar)	Mass of carbon extracted (mg)	%C extracted
**52809**	GERM01	pure lead white	19.6	106.4	0.27	1.4
**52810**	GERM02	mixture of lead white and green copper-based pigment	21.1	173	0.47	2.2
**52812**	GERM04	presence of lead	26.4	157.3	0.42	1.6
**54647**	FRIB14	lead white	15.46	152.1	0.41	2.7
**54649**	FRIB16	small amount of lead white	6.21	21.4	0.03*	0.5
**54650**	FRIB17	lead white	19.28	147.3	0.39	2
**54651**	FRIB18	lead white	12.79	76.1	0.19	1.5
**54652**	FRIB52	lead white	13.48	130.6	0.34	2.5
**54653**	FRIB53	small amount of lead white	13.00	35	0.07*	0.5

*Micro samples

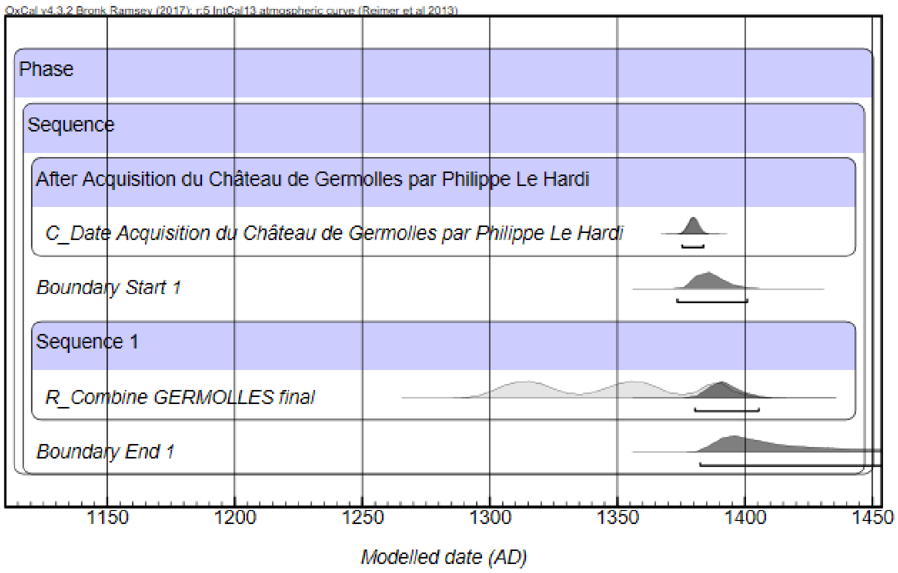

Fig. S4.
